# Feature Feedback-Based Pseudo-Label Learning for Multi-Standards in Clinical Acne Grading

**DOI:** 10.3390/bioengineering12040342

**Published:** 2025-03-26

**Authors:** Yung-Yao Chen, Hung-Tse Chan, Hsiao-Chi Wang, Chii-Shyan Wang, Hsuan-Hsiang Chen, Po-Hua Chen, Yi-Ju Chen, Shao-Hsuan Hsu, Chih-Hsien Hsia

**Affiliations:** 1 Department of Electronic and Computer Engineering, National Taiwan University of Science and Technology, Taipei 106335, Taiwan; yungyaochen@gapps.ntust.edu.tw (Y.-Y.C.); m11002113@ms.ntust.edu.tw (H.-T.C.); 2 Department of Beauty Science, National Taichung University of Science and Technology, Taichung 403027, Taiwan; 3 Department of Dermatology Division and Aesthetic Medical Center, En Chu Kong Hospital of the Hsing Tian Kong Foundation Medical Mission, New Taipei 237, Taiwan; 4 Department of Dermatology, National Taiwan University Hospital, Taipei 10002, Taiwan; psoas@ntuh.gov.tw (H.-H.C.); pohuachen@ntuh.gov.tw (P.-H.C.); yijuchen@ntu.edu.tw (Y.-J.C.); 5 Department of Dermatology, National Taiwan University Hospital Yunlin Branch, Yunlin 640, Taiwan; y05838@ylh.gov.tw; 6 Department of Computer Science and Information Engineering, National Ilan University, Yilan 260007, Taiwan; 7 Department of Business Administration, Chaoyang University of Technology, Taichung 413310, Taiwan

**Keywords:** acne, medical clinical image, deep learning, semi-supervised learning, pseudo-label, multi-standards

## Abstract

Accurate acne grading is critical in optimizing therapeutic decisions yet remains challenging due to lesion ambiguity and subjective clinical assessments. This study proposes the Feature Feedback-Based Pseudo-Label Learning (FF-PLL) framework to address these limitations through three innovations: (1) an acne feature feedback (AFF) architecture with iterative pseudo-label refinement to improve the training robustness, enhance the pseudo-label quality, and increase the feature diversity; (2) all-facial skin segmentation (AFSS) to reduce background noise, enabling precise lesion feature extraction; and (3) the AcneAugment (AA) strategy to foster model generalization by introducing diverse acne lesion representations. Experiments on the ACNE04 and ACNE-ECKH benchmark datasets demonstrate the superiority of the proposed framework, achieving accuracy of 87.33% on ACNE04 and 67.50% on ACNE-ECKH. Additionally, the model attains sensitivity of 87.31%, specificity of 90.14%, and a Youden index (YI) of 77.45% on ACNE04. These advancements establish FF-PLL as a clinically viable solution for standardized acne assessment, bridging critical gaps between computational dermatology and practical healthcare needs.

## 1. Introduction

In recent years, with the maturation of the digital era and the vigorous development of artificial intelligence (AI) technology, the application of digital transformation in the healthcare industry has become a trend, known as eHealth. Currently, the diagnosis and detection of most diseases still heavily relies on the experience and knowledge of professional clinical physicians or experts. Inevitably, this reliance may lead to some negative aspects, including variations in the clinical experience and skills of the healthcare provider. Many computer-aided detection (CADe) and computer-aided diagnosis (CADx) methods have been developed with the aim of assisting clinical physicians in making more objective and accurate diagnostic decisions. With continuous technological innovation and the increasing power of deep learning (DL) methods, medical image tasks are widely discussed and applied in various clinical diseases. These tasks encompass disease classification, disease detection, and disease segmentation.

Skin diseases rank among the most pervasive global health burdens, with epidemiological studies indicating that 70% of the worldwide population experiences at least one dermatologic condition annually [1]. Notably, cutaneous disorders constitute the fourth leading contributor to the non-fatal disease burden worldwide, as measured by the years lived with disability (YLD) metric [2]. This pervasive impact stems from diverse etiologies, including allergic, infectious, autoimmune, and neo-plastic origins, which drive both acute morbidity and chronic disability. Among dermatological conditions, acne vulgaris emerges as a high-prevalence, high-burden disease paradigm. While not life-threatening, acne affects adolescents and persists in adults, rendering it a near-universal human experience [3]. In extreme cases, individuals may exhibit inclinations toward major depressive disorder (MDD), and there have been instances of suicide that have garnered significant attention from local communities [4]. Population-level epidemiological studies reveal that individuals with moderate-to-severe acne exhibit a higher prevalence of suicidal ideation compared to the general population [5]. Notably, case–control studies have identified refractory acne as a direct contributor to psychosocial sequelae, including social withdrawal, diminished self-esteem, and impaired quality of life, which collectively escalate the risk of suicidal behavior. Clinical medical diagnosis relies heavily on the expertise and experiential knowledge of physicians, which introduces a degree of subjectivity and variability in both the methodologies and outcomes of disease diagnosis. Furthermore, annotating medical imaging data presents significant challenges, such as the high human and temporal costs involved, skewed class distributions due to the uneven prevalence of diseases, and the inherent subjectivity in physician annotations. These obstacles collectively impede the rapid assembly of a large, high-quality, and uniformly annotated medical imaging dataset. Traditional DL methodologies, when applied directly, often fail to yield significant results and are inadequate for the development of robust pre-trained weights suitable for transfer learning or federated learning paradigms.

Previous research has proposed various effective methods for acne-related tasks, such as lesion extraction and clinical grading. These methods encompass traditional hand-crafted feature-based approaches, machine learning (ML) techniques, and mainstream convolutional neural network (CNN) methods in DL. In the early stages, acne extraction tasks primarily relied on hand-crafted features and traditional ML approaches. Fujii et al. [5] identified acne features in multispectral images using linear discriminant functions (LDF). Ramli et al. [6] proposed computer vision (CV) algorithms to differentiate normal skin from acne-affected skin in images captured by digital single-lens reflex cameras. In [7], the authors extracted acne features from multispectral and thermal images. Liu et al. [8] employed Markov random fields (MRF) and chromophore descriptors for acne detection. Hayat et al. [9] utilized K-means for acne classification. Malik et al. [10] utilized K-means and support vector machines (SVM) for acne feature extraction. Alamdari et al. [11] applied fuzzy means and SVM to extract acne features and classify normal and acne-affected skin. In [12], the authors developed an acne detection system using a discrete wavelet transform (DWT) [13] and gray-level co-occurrence matrix (GLCM). In [14], the authors classified acne using a GLCM and SVM. Budhi et al. [15] performed acne segmentation and classification using region growing and a self-organizing map. Maroni et al. [16] employed random forest and Laplacian of Gaussian filtering for acne detection and lesion counting. Chantharaphaichi et al. [17] developed an acne detection system using binary thresholding. In summary, traditional CV or ML methods have provided effective means for lesion segmentation or the classification of acne in specific environments. However, common challenges include the lack of benchmark datasets for objective experimental analysis and the limited applicability of these methods to various environments, as they are often sensitive to factors such as the lighting conditions.

Regarding DL approaches, Wu et al. [18] introduced a DL framework known as label distribution learning (LDL) for severity grading and lesion counting in acne, and they released a publicly available acne grading dataset. In [19], the authors designed a CNN model for acne classification and deployed it in a mobile application. In [20], the authors created a CNN model that used attention mechanisms and dynamic context enhancement for acne detection. Lin et al. [21] employed attention mechanisms and a CNN for acne grading and conducted experiments using a self-built and non-public dataset. Pancholi et al. [22] utilized a CNN model with a regression classifier for the classification of normal skin and acne-affected skin. In summary, the commonality among these DL methods is their use of supervised learning with CNN models for classification or detection tasks. It is worth noting that the release of a public acne grading database in [18] marked a significant contribution to the field, yet there are still some common shortcomings. In particular, many of these studies lack detailed descriptions of their experimental datasets; they often use self-collected data but do not provide comprehensive details about the data, such as the annotation process; and some studies conduct experiments on self-collected datasets, which may limit the objectivity of their results.

**Figure 1:**
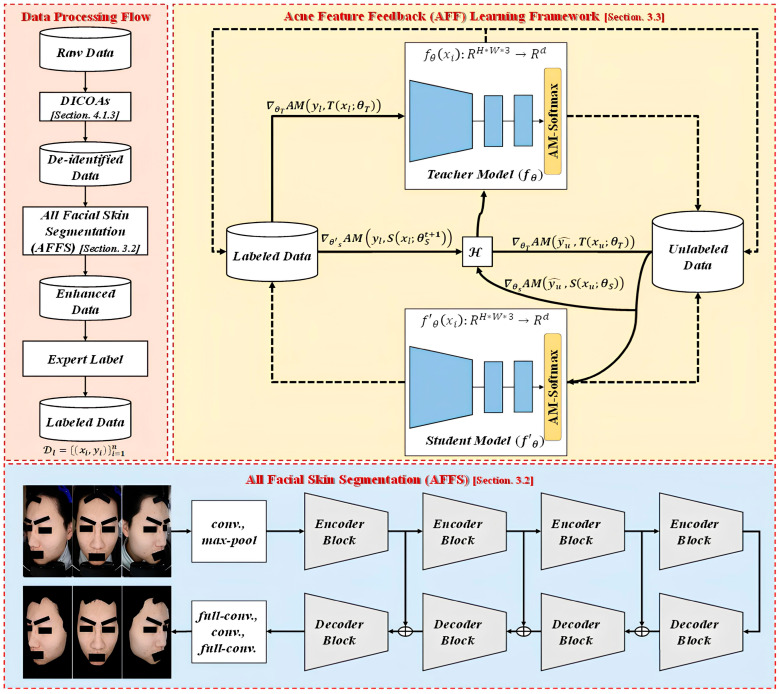
The overall framework diagram of the methodology proposed.

From traditional manual methods and DL approaches, it is evident that most techniques rely on the quantification of acne lesions or experimentation based on primary lesions. However, during actual clinical data collection and experimentation, this study found that clinical acne cases mostly involve complex lesion patterns, as illustrated in Figure 1. Therefore, it is challenging to collect data that primarily consist of simple primary lesions, as secondary changes are often a consideration. In summary, in real clinical scenarios, most acne cases involve complex lesion patterns, making it difficult for healthcare professionals to annotate each acne lesion or base their annotations solely on primary lesions. In the approach presented in this study, the consideration of secondary changes in acne provides a more clinically valuable grading system. To address the above issue, this study proposes a multi-criteria acne grading training framework. The primary contributions of this research are as follows: (1) proposing a training framework for acne grading that is adaptable to multiple grading criteria, capable of overcoming the scarcity of acne images, and reduces the dependence on annotated data; (2) Introducing a data augmentation (DA) method tailored to acne features, enabling the model to extract a wider range of lesion characteristics, enhancing its diversity, and effectively addressing complex and high-grade acne conditions; and (3) suggesting a de-identification process for acne images and establishing a database of acne grading marked by multiple dermatology experts to promote the development of AI in clinical acne grading.

## 2. Related Works

### 2.1. Acne Definition and Severity Grading Standards

Acne, also known as acne vulgaris or common acne, primarily results from the obstruction of hair follicles and sebaceous glands, which leads to an inflammatory response. The severity of acne is influenced by various factors, including age, ethnicity, climate, lifestyle, hormonal changes, squeezing, skincare routines, medications, cosmetics, skincare products, genetics, diet, sleep, stress levels, and skin characteristics such as sebum production, moisture levels, and elasticity. Acne can be categorized into five main types based on the primary lesions [23], namely comedones (blackheads and whiteheads), papules, pustules, nodules, and cystic acne. It may also exhibit secondary changes, such as inflammation, scarring, and post-inflammatory pigmentation.

Regarding the grading of acne severity, various classification systems are available in research studies and from representative organizations worldwide [24,25,26,27,28,29]. Additionally, organizations in different countries have developed different grading systems [30,31,32,33,34]. However, it is important to note that some countries and entities have not established specific grading standards, yet they continue to focus on acne treatment. In summary, while there is a relatively consistent global understanding of the primary lesions of acne, a universally accepted definition for the overall severity grading of acne remains elusive. The existence of multiple standards stems from inconsistencies among different countries, associations, or factions, resulting in a variety of criteria.

### 2.2. Self-Training in Semi-Supervised Learning

DL often leverages a substantial volume of high-quality labeled data, yielding impressive results in both theory and application. However, obtaining labeled data is far from easy and comes at a high cost. In contrast, acquiring unlabeled data is more straightforward. Hence, semi-supervised learning (SSL) has become a prominent research topic in the field of DL in recent years. SSL is a learning method that uses both labeled and unlabeled data. Compared to supervised learning, which relies solely on labeled data, SSL harnesses additional unlabeled data to enhance the learning performance, thus reducing the demand for copious labeled data. Self-training is one of the most widely adopted techniques in SSL, aiming to generate pseudo-labels for unlabeled data based on the model’s own confidence predictions.

Pseudo-labeling [35] was one of the earliest approaches to training with both labeled and unlabeled data. Typically, this type of model is trained in a supervised manner using the cross-entropy loss on labeled data. Subsequently, the same model is used for predictions on unlabeled data, and the labels with the highest-confidence predictions are referred to as pseudo-labels. Noisy Student [36] is a semi-supervised approach inspired by knowledge distillation (KD) [37] that employs a larger or equally sized student model. In terms of training, the teacher model is directly trained on labeled data while generating pseudo-labels for unlabeled data. The student model is trained with a combination of labeled and unlabeled data to create a more robust student model. During training, these samples are enhanced using RandAugment (RA), and dropout and stochastic depth are simultaneously employed in the student model. Self-supervised semi-supervised learning (S^4^L) [38] involves learning useful representations from a dataset through self-supervised learning. It calculates the loss using predicting image rotation with four angles applied in rotation [39]. It then calculates an exemplar loss using S^4^L-Exemplar to constrain the model’s stability against extensive DA. Meta Pseudo Labels (MPL) [40] reevaluates the interaction between the teacher model and student model. During model training, the student model’s performance on the validation set is fed back to the teacher model. EnAET [41] differs from semi-supervised methods. It enhances the model’s learning capacity by employing auto-encoding transformations (AET), integrating spatial transformations and non-spatial transformations to train better representations. Regarding transformations, spatial transformations include projective transformation, affine transformation, similarity transformation, and Euclidean transformation. Non-spatial transformations consist of four parameters: color, contrast, brightness, and sharpness. In terms of pseudo-label consistency, it calculates the Kullback–Leibler (KL) divergence between predictions on the original sample $\left(x\right)$ and the transformed sample $t\left(x\right)$.

SimCLRv2 [42] employs unlabeled data in a task-agnostic manner and demonstrates the substantial utility of large models in SSL. This method consists of three components: unsupervised and self-supervised pretraining, supervised fine-tuning with 1% and 10% labeled data, and self-training with task-specific unlabeled data. In terms of pretraining, it leverages the contrastive learning loss to acquire representations, where the loss function is computed based on the consistency of different augmentations of the same sample.

In summary, self-training techniques involve a series of methods to obtain pseudo-labels for unlabeled data. Pseudo-labeling uses entropy minimization to calculate and obtain high-confidence labels as pseudo-labels. Noisy Student employs various techniques during student model training, such as DA, dropout, and stochastic depth. S^4^L not only utilizes DA but also includes an additional task to enhance model performance. MPL, on the other hand, updates the teacher model’s parameters from feedback generated by the student model to improve the quality of the pseudo-labels.

### 2.3. Data Augmentation in Semi-Supervised Learning

To tackle more challenging and complex tasks, most models employ a large number of parameters and a substantial amount of data. Without an abundance of labeled data, DL models are susceptible to overfitting, resulting in poor generalization. However, acquiring labeled data can be difficult and costly, making the limitations due to limited data a prominent concern in DL models.

Traditionally, the optimal DA strategy is often designed based on heuristic algorithms or manual expertise. In contrast, AutoDA leverages a data-driven approach to discover the best augmentation strategies. Based on the method of generating augmented images, AutoDA can be further categorized into three types: (1) composition-based, (2) mixing-based, and (3) generation-based. Composition-based AutoDA is one of the most widely adopted approaches in AutoDA. It involves learning and finding the best augmentation strategies by combining multiple CV techniques. Common methods employ reinforcement learning (RL), Bayesian approaches, and gradient descent (GD). Typically, an augmentation strategy is proposed, evaluated, and updated. For instance, AutoAugment (AA) utilizes a long short-term memory (LSTM) controller to generate augmentation policies, assesses them based on the validation accuracy of its subnetwork, and employs RL for controller updates. AA involves the evaluation and updating of a large number of subnetworks, which is considered a highly resource-intensive approach (e.g., AA requires 15,000 h of GPU training to find the optimal augmentation policy on ImageNet). In comparison to AutoDA approaches using gradient descent (GD) or grid search (GS) (e.g., Differentiable Automatic DA (DADA) for GD or RA for GS), RL-based AutoDA methods (e.g., Adversarial AA, AWS AA, OHL AA) take more time to search for the optimal solution but can be more efficient in terms of GPU usage.

## 3. Proposed Feature Feedback-Based Pseudo-Label Learning

### 3.1. Overview

The framework of our proposed approach is shown in Figure 1. This work aims to establish a clinical acne grading framework. To effectively train the model with a limited amount of labeled data, we introduce the acne feature feedback (AFF) learning framework based on an SSL architecture. This framework leverages unlabeled data to address the scarcity of labeled data. To efficiently reduce background redundancy and noise in facial images, we utilize the all-facial skin segmentation (AFSS) module to extract the facial skin region of interest (ROI) images. Finally, to enhance the overall training performance, we employ AcneAugment (AA) to improve the model’s training performance. By combining these methods, the model can effectively classify acne images.

### 3.2. All-Facial Skin Segmentation

In the domain of facial skin segmentation, traditional approaches often rely on facial detection or facial tracking techniques, which require the use of facial keypoints, such as 68 or 81 key points, to capture facial features. These methods generally work well for frontal faces but often fail to capture facial features accurately in the case of profile views, making precise facial skin segmentation challenging. On the other hand, DL techniques have shown promise in achieving facial skin segmentation without the need for facial keypoints, but these methods primarily cater to frontal faces. Since common facial datasets predominantly contain frontal face images and provide relatively fewer profile face images, the direct application of existing segmentation methods on profile views may yield suboptimal results.

**Figure 2:**
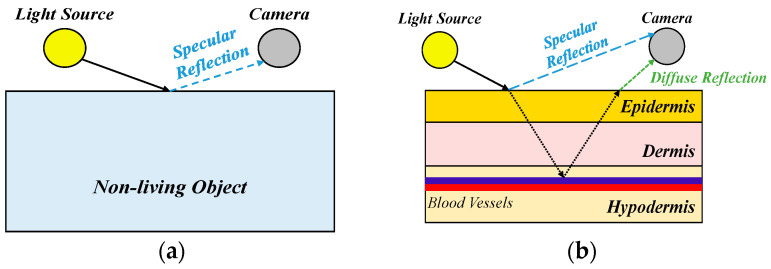
Schematic representation of rPPG signals. (**a**) non-living object; (**b**) living body.

**Figure 3:**
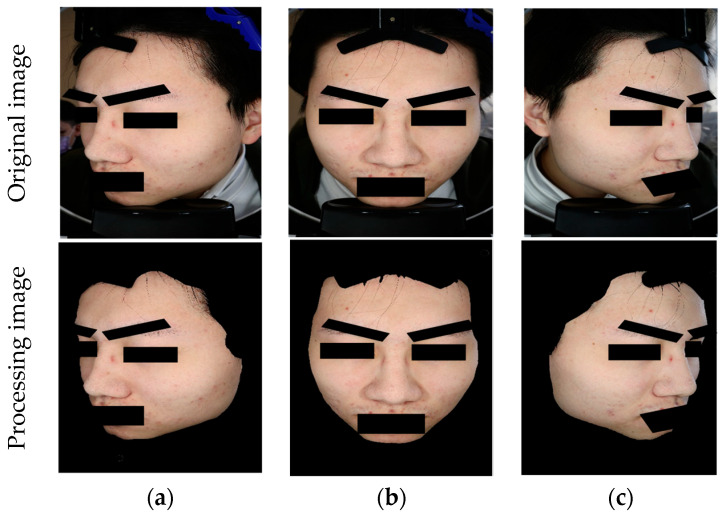
Comparison of pre-processing results before and after. (**a**) Left; (**b**) Front; (**c**) Right.

This study draws inspiration from [43], which suggests that methods based on reflectance photoplethysmography (rPPG) signals can effectively extract skin pixels, as shown in Figure 2. Consequently, we have developed a DL semantic segmentation model to distinguish skin pixels from non-skin pixels, capable of performing facial skin segmentation on both frontal and profile face images, thus addressing the limitations of traditional methods, as shown in Figure 3. To begin, we must understand the relationship between rPPG signals and the human face. rPPG methods based on facial images require the accurate identification of the skin region of the face to enable effective signal extraction. rPPG refers to the contrast in light reflection or diffusion when light illuminates the facial skin. Therefore, we can use rPPG as a basis for discriminating between the face (living tissue) and non-face (background). This distinction depends on the degree of light reflection from the skin, where some of the light is absorbed by the skin and blood vessels, while the remainder is received by the camera.

**Figure 4:**
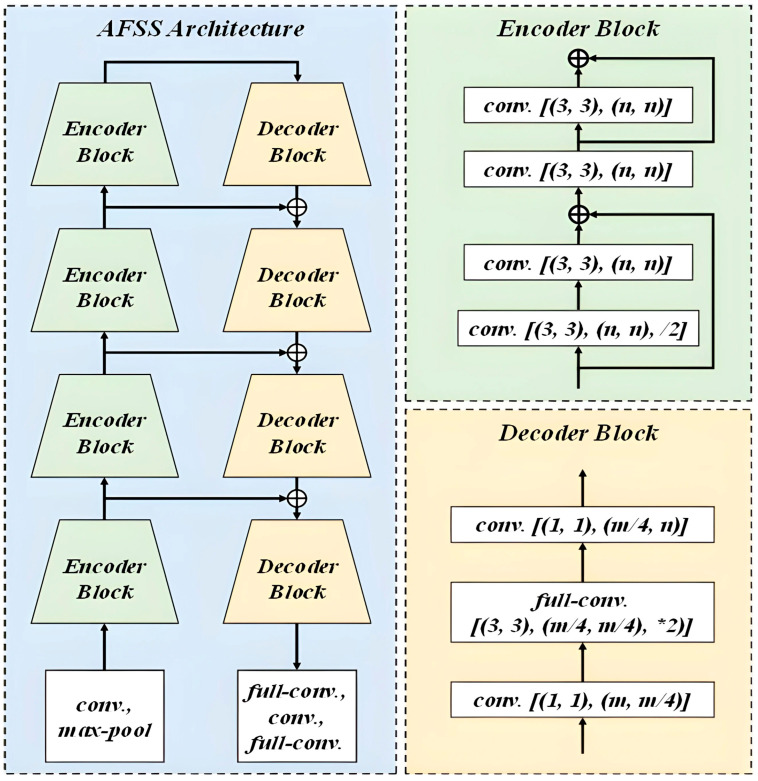
Schematic diagram of the AFSS architecture.

Regarding the segmentation model, we have adapted the LinkNet [44] model, known for its ability to achieve good training results on relatively limited profile face skin datasets. Its simple architecture is highly adaptable and applicable to various tasks in different domains. Specifically, the AFSS architecture is an encoder–decoder structure, as illustrated in Figure 4. In Table 1, each encoder and decoder block consists of a CNN for the extraction of deep semantic information. On the other hand, since the process of feature extraction is prone to losing fine-grained details, we have incorporated link propagation in AFSS, including skip connections and feature fusion, to transmit information between the encoder and decoder. This approach has the advantage of compensating for lost fine-grained features and ensuring the retention of crucial semantic information at different levels.

### 3.3. Acne Feature Feedback Learning Framework

**Figure 5:**
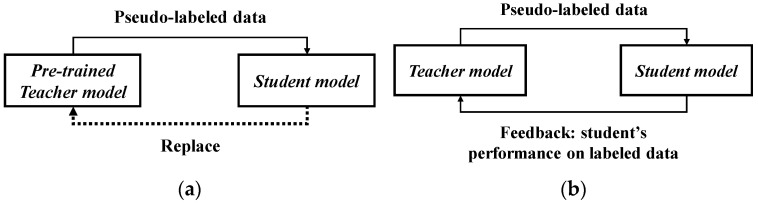
Comparison between traditional pseudo-label method and feedback-based pseudo-label method. (**a**) Traditional pseudo-label method; (**b**) feedback-based pseudo-label method.

For the acne grading task in medical image analysis, data collection often presents significant challenges due to the following factors: (1) privacy concerns and variations in disease severity make data collection difficult; (2) time and labor costs make data annotation a non-trivial task. These circumstances result in a severe shortage of acne data for training, rendering traditional supervised learning methods, which rely on abundant labeled data, impractical for this task. Therefore, this study proposes the AFF learning framework within an SSL framework, addressing these limitations. The advantages of this framework are as follows: (1) it effectively reduces the dependency on a large amount of annotated acne data—during the training process, in addition to labeled data, it concurrently learns from unlabeled data, diversifying the extracted features; (2) it caters to scenarios characterized by high inter-class similarity and intra-class variability in acne features; (3) by utilizing AM-Softmax, it enhances the inter-class feature distances while minimizing the intra-class distances, thereby improving the separability of different classes and the compactness of features within the same class. Generally, SSL with pseudo-labeling involves a network comprising a teacher model and a student model. During training, the teacher model generates pseudo-labels on unlabeled data, which are then passed to the student model for learning. By utilizing a large amount of unlabeled data and regularization techniques, the trained student model typically outperforms the teacher model. However, it has a critical drawback: if the pseudo-labels generated by the teacher model are incorrect, the training of the student model can deteriorate, leading the overall model training in a negative direction, a phenomenon known as cognitive bias. To address this issue, this study redesigns the training mechanism, enabling the teacher model to observe the learning progress of the student model (See the Algorithms 1). In other words, the learning effectiveness of the student model is fed back to the teacher model, allowing the teacher model to generate more accurate pseudo-labels based on this feedback, thereby enhancing the entire learning process, as shown in Figure 5. This approach mitigates the cognitive bias introduced by incorrect pseudo-labels and improves the model’s performance and stability.

In terms of the forward and backward computations during the model training process, the teacher model infers unlabeled data, generating pseudo-labels, and subsequently converts them into a hard label format. The student model then undergoes training based on the pseudo-labels generated by the teacher model, which can be represented as follows:

(1) $$\nabla_{\theta_{s}}AM\left(\hat{y_{u}}\;,S\left(x_{u};\theta_{S}\right)\right)$$

Subsequently, the student model infers labeled data and computes gradients during the backward pass, which can be represented as follows:

(2) $$\nabla_{{\theta^{\prime }}_{s}}AM\left(y_{l},S\left(x_{l};\theta_{S}^{t+1}\right)\right)$$

Additionally, the previously obtained gradients are reutilized to further compute the feedback value (H) for the student model through an inner product. The teacher model proceeds to predict unlabeled data once more and calculate gradients. Since the pseudo-labels have been transformed into hard labels during the initial step, the loss function can be directly applied for computation, which can be represented as follows:

(3) $$\nabla_{\theta_{T}}AM\left(\hat{y_{u}}\;,T\left(x_{u};\theta_{T}\right)\right)$$

This gradient will be multiplied by (H). Finally, the teacher model also predicts labeled data and computes gradients, which can be represented as

(4) $$\nabla_{\theta_{T}}AM\left(y_{l},T\left(x_{l};\theta_{T}\right)\right)$$

Combining these steps allows for a comprehensive gradient update, which can be represented as

(5) $$\nabla_{\theta_{T}}L_{l}=\eta s\cdot H\cdot \nabla_{\theta_{T}}AM\left(y_{l},T\left(x_{l};\theta_{T}\right)\right)\;=\eta s\cdot \left[\nabla_{{\theta^{\prime }}_{s}}AM\left(y_{l},S\left(x_{l};\theta_{S}^{t+1}\right)\right)\cdot \nabla_{\theta_{s}}AM\left(\hat{y_{u}}\;,S\left(x_{u};\theta_{S}\right)\right)\right]\cdot \nabla_{\theta_{T}}AM\left(y_{l},T\left(x_{l};\theta_{T}\right)\right)$$

Regarding the interaction between the teacher model and the student model, the part where the teacher model generates pseudo-labels to train the student model can be directly viewed as the minimization of the loss on unlabeled data associated with pseudo-labels, which can be represented as

(6) $$\theta_{S}^{PL}={argmin}_{\theta_{S}}\;E_{x_{u}}\left[AM\left(T\left(x_{u};\theta_{T}\right),S\left(x_{u};\theta_{S}\right)\right)\right]$$

In this context, the pseudo-label target $T\left(x_{u};\theta_{T}\right)$ is generated by a well-trained teacher model with fixed parameters $\theta_{T}$ and is subject to the condition $E_{x_{u}}\left[CE\left(T\left(x_{u};\theta_{T}\right),S\left(x_{u};\theta_{S}\right)\right)\right]∶=L_{u}\left(\theta_{T},\theta_{S}\right)$.

The part in which the student model utilizes its inference performance on labeled data as feedback to refine the teacher model can be represented as

(7) $${min}_{\theta_{T}}L_{l}\left(\theta_{S}^{PL}\left(\theta_{T}\right)\right),where\;\theta_{S}^{PL}\left(\theta_{T}\right)={argmin}_{\theta_{S}}\;L_{u}\left(\theta_{T},\theta_{S}\right)$$

Combining the two equations above, it is evident that Equations (6) and (7) exhibit a coupling relationship, making the optimization process significantly complex and computationally challenging. Hence, it becomes necessary to expand the expression of the student model influenced by the teacher model, rendering it amenable to straightforward gradient updates. Specifically, $\theta_{S}^{PL}\left(\theta_{T}\right)\approx \theta_{S}-\eta s\cdot \nabla_{\theta_{s}}L_{u}(\theta_{T},\theta_{S})$. Therefore, the previous representation should be rewritten as

(8) $${min}_{\theta_{T}}L_{l}\left(\theta_{S}-\eta s\cdot \nabla_{\theta_{s}}L_{u}(\theta_{T},\theta_{S})\right)$$

After discussing the forward and backward propagation of the model and its interaction dynamics, we will now elucidate the importance of the loss function within this framework, which plays a pivotal role in this task. The decision boundary of the loss function affects the classification performance of feature groups and can have a substantial impact on the training regime when using unlabeled data, determining whether the training is prone to instability. The key lies in the ability to adjust the distances between features. Taking the widely used Softmax as an example, it employs the angular margin to determine an additive margin, namely cos-m. Specifically, AM-Softmax can be derived from the classical Softmax. The basic Softmax can be represented as

(9) $$L_{S}=-\frac{1}{n}\sum_{i=1}^{n}\log\frac{e^{W_{y_{i}}^{T}f_{i}}}{\sum_{j=1}^{c}e^{W_{j}^{T}f_{i}}}=-\frac{1}{n}\sum_{i=1}^{n}\log\frac{e^{\left\|W_{y_{i}}\right\|\left\|f_{i}\right\|\cos\left(\theta_{y_{i}}\right)}}{e^{\left\|W_{j}\right\|\left\|f_{i}\right\|\cos\left(\theta_{j}\right)}}$$

where (f) represents the input to the final fully connected layer, $f_{i}$ denotes the *i*th sample, $W_{j}$ is the *j*th column of the final fully connected layer, and $W_{y_{i}}^{T}f_{i}$ is the target logit for the *i*th sample. In the A-Softmax loss, the weight vectors are normalized by setting $\left\|W_{j}\right\|$ to 1, and the target-logit is transformed from $\left\|f_{i}\right\|\cos\left(\theta_{y_{i}}\right)$ to $\left\|f_{i}\right\|\psi \left(\theta_{y_{i}}\right)$. Therefore, the A-Softmax loss can be expressed as

(10) $$L_{AS}=-\frac{1}{n}\sum_{i=1}^{n}\log\frac{e^{\left\|f_{i}\right\|\psi \left(\theta_{y_{i}}\right)}}{e^{\left\|f_{i}\right\|\psi \left(\theta_{y_{i}}\right)}+\sum_{j=1}^{c}e^{\left\|f_{i}\right\|\cos\left(\theta_{j}\right)}}$$

where $\psi \left(\theta \right)$ represents a piece-wise function and can be defined as

(11) $$\psi \left(\theta \right)=\frac{{\left(-1\right)}^{k}\cos\left(m\theta \right)-2k+\lambda \cos\left(\theta \right)}{1+\lambda }\;,\theta \in \left[\frac{k\pi }{m},\frac{(k+1)\pi }{m}\right]$$

where (m) is an integer greater than 1, and (λ) is a hyperparameter used to control the degree of the classification boundary.

Inspired by both Softmax and A-Softmax, AM-Softmax normalizes the input of feature weight vectors as $x=\cos\left(\theta_{y_{i}}\right)=\frac{W_{y_{i}}^{T}f_{i}}{\left\|W_{y_{i}}\right\|\left\|f_{i}\right\|}$. Therefore, during the forward propagation of model training, only the following needs to be applied:

(12) $$\Psi \left(x\right)=x-m$$

This advantage eliminates the need for computing additional backpropagation gradients, making it more programmatically straightforward to implement. On the other hand, since the cosine similarity is employed, the features and weight normalization are applied to the inner product for the construction of the cosine layer. Then, scaling with (s) is used to adjust the cosine. Therefore, the final AM-softmax loss can be expressed as

(13) $$L_{AM}=-\frac{1}{n}\sum_{i=1}^{n}\log\frac{e^{s\cdot \left(\cos\left(\theta_{y_{i}}\right)-m\right)}}{e^{s\cdot \left(\cos\left(\theta_{y_{i}}\right)-m\right)}+\sum_{j=1,j\neq y_{i}}^{c}e^{s\cdot \cos\left(\theta_{j}\right)}}\;=-\frac{1}{n}\sum_{i=1}^{n}\log\frac{e^{s\cdot \left(W_{y_{i}}^{T}f_{i}-m\right)}}{e^{s\cdot \left(W_{y_{i}}^{T}f_{i}-m\right)}+\sum_{j=1,j\neq y_{i}}^{c}e^{s\cdot W_{y_{i}}^{T}f_{i}}}$$

The architecture of SSL demands greater computational resources for both forward and backward propagation compared to standard supervised learning. Therefore, this framework requires a loss function that does not significantly impact the computational resources and can effectively handle inter-class similarity and intra-class variability. AM-Softmax fits this requirement perfectly as it does not necessitate additional backpropagation gradient computations, offering substantial improvements with only slight modifications to the classic Softmax.
**Algorithm 1.** Pseudocode for acne feature feedback learning framework.1:Input: Labeled data $\left(x_{l},y_{l}\right)$ and unlabeled data $\left(x_{u}\right)$.2:Initialize $\left(\theta_{T},\theta_{S}\right)$.3:for (t=0) to (N−1) do4: Sample an unlabeled example $\left(x_{u}\right)$ and a labeled example $\left(x_{l},y_{l}\right)$5: Sample a pseudo-label $\hat{y_{u}}\sim P\left(\cdot |x_{u};\theta_{T}\right)$6: Update the student using the pseudo-label $\hat{y_{u}}$:7:$\theta_{S}^{t+1}=\theta_{S}^{t}-\eta s\cdot \nabla_{\theta_{s}}E\left(\hat{y_{u}},S\left(x_{u};\theta_{S}^{t}\right)\right)$8: Compute the teacher’s feedback coefficient:9:$\eta s\cdot \left[\nabla_{{\theta^{\prime }}_{s}}AM\left(y_{l},S\left(x_{l};\theta_{S}^{t+1}\right)\right)\cdot \nabla_{\theta_{s}}AM\left(\hat{y_{u}},S\left(x_{u};\theta_{S}^{t}\right)\right)\right]$10: Compute the teacher’s gradient from the student’s feedback:11:$g_{T}^{\left(t\right)}=h\cdot \nabla_{\theta_{T}}AM\left(\hat{y_{u}},T\left(x_{u};\theta_{T}^{t}\right)\right)$12: Compute the teacher’s gradient on labeled data:13:$g_{T,supervised}^{\left(t\right)}=\nabla_{\theta_{T}}AM\left(y_{l},T\left(x_{l};\theta_{T}^{t}\right)\right)$14: Compute the teacher’s gradient on the UDA loss with unlabeled data:15:$g_{T,UDA}^{\left(t\right)}=\nabla_{\theta_{T}}AM\left(StopGradient\left(T\left(x_{l}\right);\theta_{T}^{t}\right),T\left(AcneAugment\left(x_{l}\right);\theta_{T}^{t}\right)\right)$16: Update the teacher:17:$\theta_{T}^{t+1}=\theta_{T}^{t}-\eta T\cdot \left(g_{T}^{\left(t\right)}+g_{T,supervised}^{\left(t\right)}+g_{T,UDA}^{\left(t\right)}\right)$18:End19:Return $\theta_{S}^{N}$

### 3.4. AcneAugment (AA)

DA is one solution to address the problem of limited training data by enhancing the data diversity and quantity through random transformations. However, the design of augmentation strategies often requires domain-specific knowledge to capture the prior knowledge of each application area. This makes it challenging to directly extend traditional DA methods to other application domains. Recently, auto-designed augmentation methods based on learning strategies (e.g., AA, Fast AA, RA) have been able to overcome some limitations of traditional DA methods. Therefore, in this study, we adopt an auto-designed augmentation strategy.

In this paper, we propose an acne-specific DA method called AA based on RA. It is a GS-based AutoDA, with a search space of only 10^2^, making it more efficient in terms of GPU time compared to common RL-based AutoDA approaches. Acne features are highly sensitive to color variations, which can lead to misclassification. Therefore, we excluded some related augmentation methods, including color, contrast, invert, posterize, and sharpness, but retained auto-contrast, which performs automatic adjustments and has a positive impact on the model’s overall training. We also retained the brightness, which we found to have a positive learning effect on skin oil reflection. On the other hand, the overlapping or mixing of acne features can have a negative impact on model training, so we excluded related augmentation methods, including simple pairing. Through these strategies, we ensure that the characteristics of acne lesions remain invariant, making the model training process more stable and enhancing its generalization ability, with the detailed parameters as shown in Table 2. In addition, the robust training process ensures that the model with the semi-supervised architecture does not learn in the wrong direction, preventing the training from breaking down.

## 4. Experiments and Results

### 4.1. Datasets

This study proposes two different standard acne grading datasets, namely ACNE04 and ACNE-ECKH, to validate the effectiveness of the proposed method. The de-identification process has been validated to ensure patient anonymity. A detailed comparison of these datasets is shown in Table 2, Table 3 and Table 4.

**Figure 6:**
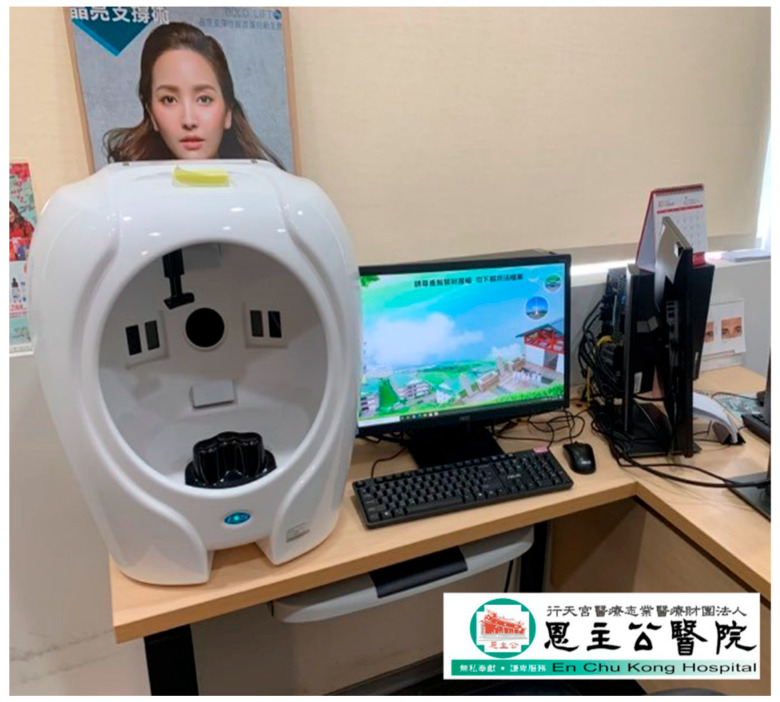
Photography equipment set up inside the hospital examination room in this study.

**Figure 7:**
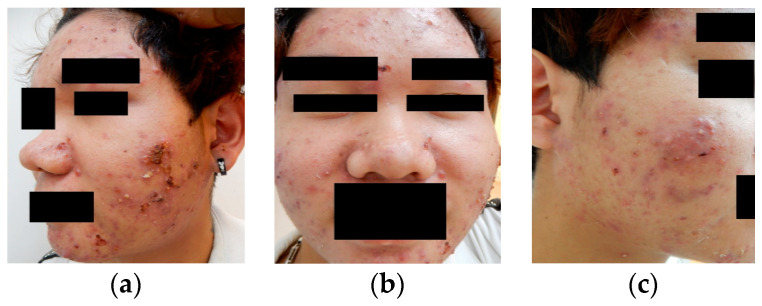
Illustration demonstrating the insufficiency of a single orientation in representing all acne conditions. (**a**) Left, (**b**) front, and (**c**) right view.

The ACNE04 dataset is the first publicly available dataset for acne grading research [18]. It consists of 1457 images and follows the acne grading criteria proposed by Hayashi [33], which categorize acne into four different grades. The dataset has been annotated by medical professionals. The ACNE-ECKH dataset was collected in a clinical environment at a regional hospital, with the involvement of specialized medical professionals and equipment. Patients aged 12–50 years who visited En Chu Kong Hospital for acne were enrolled in this study. The inclusion criteria for subjects were a diagnosis of acne by a dermatologist and a Dermatology Life Quality Index (DLQI). This non-invasive study was open to all patients, and informed consent forms were signed (IRB No. ECKIRB1101002). Multiple dermatologists graded the acne lesions into four levels according to the Bernardis standard [29]. This dataset comprises 8242 images for acne image classification, consisting of 1500 labeled images and 6742 unlabeled images. Regarding the labeled images, digital equipment, as depicted in Figure 6, was used to capture the images. Each patient had three images taken, as a single direction was insufficient to fully represent the acne condition of each patient (Figure 7). Hence, each patient required photographs of their left profile, frontal view, and right profile. The angles between the left and right profiles were approximately 33 degrees. Patients were instructed to cleanse their entire face before photography, ensuring a clear and unobstructed view. The patients in this dataset were of Asian descent, with skin tones ranging from Fitzpatrick scale levels II to IV. It is worth noting that all images in this dataset underwent a de-identification process to ensure patient privacy and data security.

For the unlabeled images, due to the rarity of the condition, data security and privacy concerns, and the absence of proper medical expert annotations, accumulating a large quantity of high-quality and consistent clinical data within a short timeframe was challenging. Therefore, the creation of unlabeled data is essential to reduce the reliance on the quantity of medical imaging data for DL. Additionally, it can enhance the feature diversity for more complex lesions. To address these challenges, this study utilized web scraping techniques to obtain 6742 images containing acne. The scraping process involved using “acne” as a keyword, and the keyword was translated into multiple languages (e.g., English, French) to perform web scraping, resulting in the collection of thousands of images.

For DL, the dataset’s quality plays a pivotal role in the success of the model. In the domain of medical imaging tasks, there is a clear trend towards establishing large datasets, which not only drive technological advancements but also facilitate improvements in human health. However, as medical images grow in volume and become accessible on the internet, safeguarding patient privacy becomes an essential consideration. In view of this, our study proposes a de-identification process tailored to acne patients, with the goal of providing a globally accepted approach to medical image creation, ultimately benefiting overall human health.

**Figure 8:**
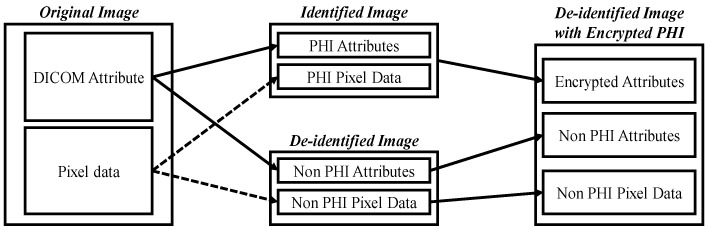
De-identification system for digital acne images.

The purpose of de-identification is to ensure the safe handling of protected health information (PHI) and patient privacy when DICOM is used in secondary applications or image exchange scenarios, reducing the risk of re-identification. Figure 8 illustrates the de-identification process applicable to acne patients, developed based on the HIPAA, CoE 108 Convention, and MISA-TW guidelines. Specifically, the image part involves completely obscuring the eyes, eyebrows, and mouth with black rectangles to remove identifiable visual features. In the case of file names, they are first converted to ASCII encoding and then hashed using the MD5 hash function. In summary, this process contributes to the creation and sharing of medical imaging datasets, with the overarching goal of promoting the advancement of medical imaging tasks for the betterment of human well-being.

### 4.2. Evaluation Metrics

This work uses common classification performance metrics, including accuracy and precision, along with three representative evaluation metrics from the medical domain, namely sensitivity, specificity, and the Youden Index (YI), to assess the model’s performance. Accuracy is an evaluation metric reflecting the overall model performance, representing the proportion of samples correctly predicted by the model to the total number of samples. It is calculated as follows:

(14) $$\mathrm{Accuracy}\left(Acc.\right)=\frac{TP+TN}{TP+TN+FP+FN}=\frac{N_{c}}{N}$$

where $N_{c}$ denotes the number of samples correctly predicted by the model, and N is the total number of samples predicted by the model.

Precision measures the proportion of correct diagnoses, with higher precision indicating the more accurate diagnosis of the target disease, as shown in Equation (15). Sensitivity represents the proportion of correctly detected positive results among patients who have the disease, calculated according to Equation (16). Specificity measures the proportion of correctly detected negative results among patients who do not have the disease, calculated as in Equation (17). The YI provides a comprehensive assessment of the disease diagnosis capability, with a higher Youden index indicating good performance in both the correct diagnosis and exclusion of the disease, as shown in Equation (18). For all evaluation metrics, *TP*, *FP*, *TN*, and *FN* represent true positives, false positives, true negatives, and false negatives, respectively.

(15) $$Precision\left(Pre.\right)=\frac{TP}{TP+FP}$$

(16) $$Sensitivity\left(Se.\right)=\frac{TP}{TP+FN}$$

(17) $$Specificity\left(Sp.\right)=\frac{TN}{TN+FP}$$

(18) Youden Index(YI)=Sensitivity+Specificity−1

### 4.3. Implementation Details

Based on our experience in CV and DL tasks, we configured the hyperparameters with the following settings. All experiments were conducted on a computer equipped with an NVIDIA GeForce RTX 3090 GPU (Santa Clara, CA, USA)and an Intel i7-10700 CPU (Santa Clara, CA, USA), utilizing the PyTorch 2.1.0 and TensorFlow DL frameworks. The program implementation involved referencing and modifying open-source codebases such as PyTorch Image Models (TIMM), UDA, MPL, Kappa, and LinkNet for Facial Skin [45].

In the context of hyperparameters, the system can be divided into two categories: supervised learning and pseudo-label learning. As shown in Table 5, in the supervised learning section, both the ACNE-ECKH and ACNE04 backbones are updated using the Nesterov momentum, with an initial momentum coefficient of 0.9. The model’s learning rate is subject to cosine decay, with an initial value of 0.01. Due to experimental constraints, in this research, the batch size was set to 8 and 16. Regarding the number of training iterations, we adhered to common practice, setting it to 50,000 and employing warm-up steps to gradually reduce the learning rate, with a value of 2500.

As shown in Table 6, for pseudo-label learning, training is performed using RMSProp and an exponential decay learning rate schedule. It is worth noting that the reason for not using the commonly employed SGD for fine-tuning is that the student model does not directly learn from labeled data, allowing for the use of more powerful optimizers. Typically, in state-of-the-art methods, for standard datasets (e.g., CIFAR-10, ImageNet), common batch sizes include 1024 or 128. Due to limitations in our experimental environment, we set the batch size to 8. Regarding the number of training iterations, we followed standard practice, setting it to 3,000,000 and employing warm-up steps to gradually reduce the learning rate, with a value of 2000.

### 4.4. Results

Visual Transformers, like ViT and Swin Transformer, offer significant advancements in image understanding but have notable limitations. Firstly, they require large-scale datasets and extensive pretraining to achieve competitive performance, making them resource-intensive. Secondly, their computational complexity and memory consumption increase quadratically with the image size, leading to challenges in scalability. Thirdly, their dependency on extensive fine-tuning for specific tasks can limit generalization. Thus, we validated the effectiveness of our proposed method across datasets with different standards in this study. We conducted experiments on two datasets: ACNE04 using the Hayashi standard and ACNE-ECKH using the Bernardis standard. We used the precision, accuracy, sensitivity, specificity, and YI as the unified evaluation metrics. Firstly, in the case of the ACNE04 dataset, we compared our proposed method with three categories of approaches, including LDL methods, traditional manual methods, and DL methods, as shown in Table 7. The LDL methods included PT-Bayes, PT-SVM, AA-KNN, AA-BP, SA-IIS, SA-BFGS, and SA-CPNN. The manual feature-based methods encompassed HOG, SIFT, GABOR, and CH. The DL methods included VGG, ResNet, SE-ResNet, and EfficientNet models, as well as state-of-the-art methods on the ACNE04 dataset, such as LDL, KIEGLFN, EGPJ, and DED.

From Table 7, it is evident that the manual feature-based methods exhibit the overall poorest performance. The diverse appearances of acne lesions due to primary and secondary lesions, as well as factors like lesion rupture, cause variations in the acne’s appearance. Manual feature-based methods are unable to effectively adapt to the diversity of acne lesions, rendering them of limited diagnostic value. In contrast to manual feature-based methods, DL methods leverage deep semantic features and DA techniques, making it easier to train robust models. The DL methods performed the best in terms of overall performance. Our proposed method is also based on DL and outperforms the SOTA methods, with the highest precision (88.25%) and accuracy (87.33%). It also demonstrates good sensitivity (87.31%), specificity (90.14%), and YI values (77.45%).

LDL methods, which are hybrid approaches, combine aspects of DL models with label distribution definitions. They require the labeling of each acne severity level for each acne instance, which may not be practical in real clinical settings due to the difficulty of marking every acne lesion. Many clinical cases involve complex acne lesion scenarios, mainly driven by secondary lesions, and multiple factors, making it impractical to label every lesion. As a result, while these methods perform well on the ACNE04 dataset, the extensive labeling requirements hinder their practicality.

Table 8 presents a comparison between our approach and DL methods across two different standard datasets. The results indicate that the classical SOTA CNN models, initially designed for natural image datasets like CIFAR and ImageNet, cannot be directly applied to skin lesion tasks, with peak accuracies of only 80.46% for ACNE04 and 37.5% for ACNE-ECKH. This discrepancy can be attributed to significant differences in the dataset scale and labeling complexity. The CIFAR and ImageNet datasets are several orders of magnitude larger, whereas ACNE04 and ACNE-ECKH consist of only a few thousand samples. Building medical image datasets is more challenging, and DL methods must be adapted to accommodate these differences. Consequently, our approach adopts a semi-supervised framework, allowing models to learn more lesion diversity from unlabeled data and better adapt to the rich variety of acne lesions. By utilizing a DA method tailored to acne lesions, our model can more effectively learn these variations, leading to impressive accuracies of 87.33% for ACNE04 and 67.50% for ACNE-ECKH.

Our proposed approach is less affected by the quantity of labeled data and the labeling costs and can adapt well to lesions with diverse characteristics. From the overall performance shown in Table 7 and Table 8, the performance on ACNE04 surpasses that on ACNE-ECKH. The primary factor contributing to this discrepancy is the difference in the distribution of the test dataset, as indicated in Table 3. The ACNE04 test dataset has uneven class distributions, leading to biases in model testing. Models tend to perform better in classes with more instances, resulting in higher accuracy. In contrast, the ACNE-ECKH test dataset has equal class distributions for all categories, offering more objectivity in model testing and avoiding any bias towards a specific class. For example, regarding the ACNE04 dataset, when the test set constituted 10% of the overall dataset, the model achieved accuracy of 89.12%, sensitivity of 88.45%, and specificity of 91.23%. When the test set is relatively small, such as 10%, it generally exhibits higher accuracy due to lower sample variability. For a test set comprising 20% of the data, which corresponded to our original data partition, the model demonstrated accuracy of 87.33%, sensitivity of 87.31%, and specificity of 90.14%. This partitioning strategy was employed in our study to ensure a balanced distribution between the training and test sets. When the test set accounts for 30% of the data, the model’s performance declines slightly, with accuracy of 85.67%, sensitivity of 85.12%, and specificity of 88.89%. Although a larger test set, such as 30%, provides a more rigorous evaluation, the increased data diversity may lead to a modest reduction in the performance metrics. Overall, despite the variations in the test set proportions, the model’s performance remains stable, with an accuracy fluctuation within ±3%, indicating the robust generalizability of our framework. Furthermore, we ensured that there was no patient overlap between the training and test sets, thereby preventing data leakage and ensuring the validity of clinical evaluations. We chose 20% as the test set for this work.

### 4.5. Ablation Study

#### 4.5.1. Comparison Between Different Modules

To validate the effectiveness of the various components used in our proposed method, we conducted experiments comparing each module of the proposed method, as shown in Table 9 and Table 10. Initially, we performed an analysis of the results for the ACNE-ECKH dataset experiment. When no modules were used, indicating the utilization of standard supervised learning (with EfficientNet-L2 as the backbone), the accuracy was 37.50%, as shown in Table 9. However, when we implemented the SSL framework, the accuracy was significantly improved to 42.50%, representing an overall increase of 5% compared to supervised learning.

Subsequently, we conducted detailed comparisons between the two main modules. All experiments were carried out within the SSL framework, considering the results achieved with either a single module or both modules. According to the experimental results in Table 9, using the AFSS module alone led to accuracy of 50.00%, which is a 12.50% overall improvement, while using the AA module alone led to a 10.00% overall improvement. The analysis of the individual modules suggests that the AFSS module, employed for the elimination of background noise, produces the most substantial results, while the AA module, which focuses on increasing the data diversity, also yields significant benefits. Moreover, we conducted a comprehensive comparison of the results achieved with the combination of both modules. The combination of the AFSS and AA modules performed the best, resulting in accuracy of 67.25%, representing an overall improvement of 30.00%.

In addition to the experiments on the ACNE-ECKH dataset, we conducted experimental analyses on the ACNE04 dataset, as presented in Table 10. The results reveal that, without the use of any modules, relying solely on standard supervised learning (with EfficientNet-L2 as the backbone), the accuracy was 80.13%. However, when we adopted the SSL framework, the accuracy increased to 83.21%, representing an overall improvement of 3.08% compared to supervised learning. Subsequently, we conducted detailed comparisons considering whether to use the two main modules, both within the context of an SSL experiment. As the results showed, using the AFSS module alone resulted in accuracy of 84.24%, which was a 4.11% overall improvement, whereas using the AA module alone led to accuracy of 85.95%, a 5.82% overall improvement.

The analysis of the individual modules revealed that the AFSS module did not perform as well with the ACNE04 dataset, producing only a 4.11% improvement when compared to the results in the previous section. We believe that this discrepancy may be attributed to the significant differences between the ACNE04 and the ACNE-ECKH datasets, as detailed in Table 4. The ACNE04 dataset contains several low-quality images, whereas the ACNE-ECKH dataset consists exclusively of images collected from hospitals and maintains a certain standard.

#### 4.5.2. Comparison Between Different Types of Facial Skin Segmentation

To demonstrate the finer skin segmentation capabilities of the AFSS module proposed in this study, we conducted visual comparisons between AFSS and other segmentation methods on two acne grading datasets, namely ACNE04 and ACNE-ECKH. The alternative methods used for comparison included Haar-cascade, GrabCut, and keypoint. The experiments were all performed on the original dataset images at the same resolution, with variations in the processing approach.

**Figure 9:**
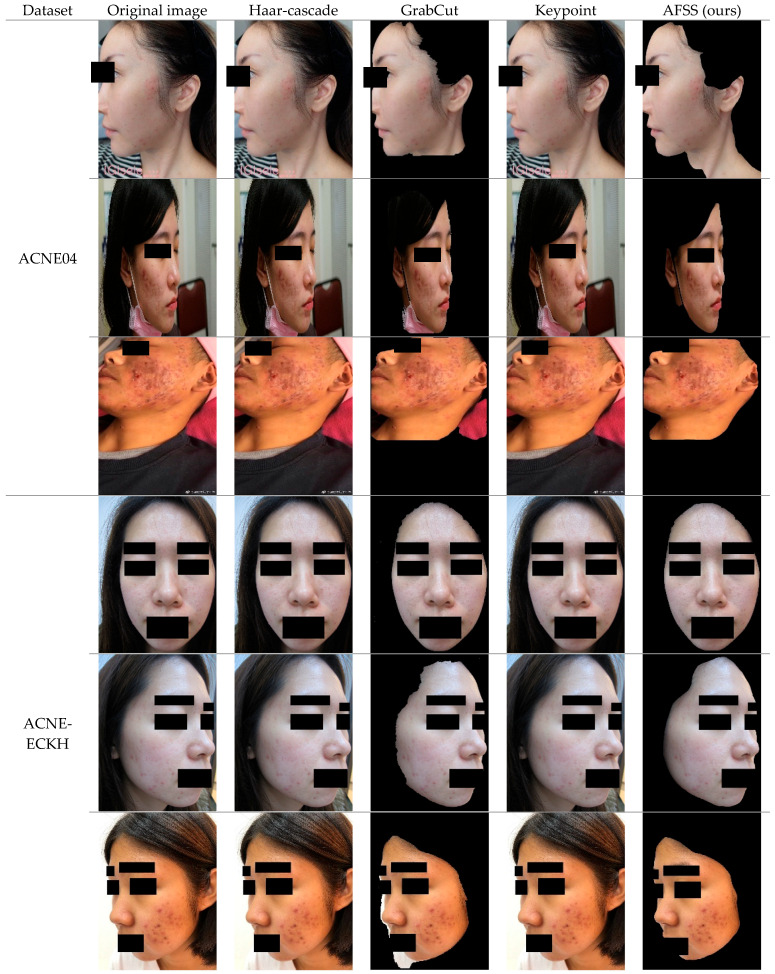
Comparison between different facial skin segmentation methods on two datasets.

The segmentation results obtained from the two acne grading datasets indicate that common methods like Haar-cascade and keypoint, which are based on local features, can only effectively process samples with well-defined facial features, as shown in Figure 9. They struggle to handle samples with unclear facial features, such as side views, closed eyes, and de-identified images. In summary, these methods are limited not only by the clarity of facial features but also by specific sample environments, resulting in suboptimal skin segmentation for both frontal and profile views. In contrast, both AFSS and GrabCut are pixel distribution-based methods that can effectively segment facial skin in both frontal and profile samples. Compared to GrabCut, AFSS provides even finer segmentation results and can exclude non-human pixels, including masks and background elements.

Additionally, we provide several lines of evidence that the proposed approach indeed extracts useful and discriminative image features, rather than merely capturing noise. Table 9 and Table 10 illustrate that, when the AFSS module is incorporated, the accuracy improves significantly compared to the baseline supervised method. This indicates that AFSS effectively suppresses irrelevant background noise while emphasizing the salient facial skin features that are crucial for acne grading. Furthermore, the combination of AFSS with our AA module further enhances the performance, resulting in overall accuracy improvements on certain datasets. This supports the assertion that the network learns robust and useful features, rather than random noise.

#### 4.5.3. Comparison Between Different Auto-Augment Methods

We conducted a comparison between AA and other automated augmentation methods on the two acne grading datasets, ACNE04 and ACNE-ECKH. The alternative methods included AA and RA based on the CIFAR/ImageNet/SVHN datasets. The experiments were all based on the methods proposed in this paper, with differences in the use of DA strategies and the specific augmentation techniques employed.

In Table 11, the results for both acne grading datasets indicate that AA performs exceptionally well in all metrics, showcasing superior diagnostic capabilities and good accuracy in disease diagnosis. Compared to the second-best performer, RA (CIFAR/ImageNet), AA outperforms it by excluding color-related transformations (e.g., color, contrast) and methods causing overlapping or the confusion of lesion features (e.g., sample pairing). RA (CIFAR/ImageNet) shows slightly lower performance but incorporates three additional transformation methods, namely sample pairing, rotate, and translate-x. Rotate and translate-x are geometric transformations that do not cause color variations, leading to some performance improvement. However, sample pairing results in the overlapping or confusion of lesion features, causing a drop in the learning performance as overlapped lesions form new complex lesions.

The results obtained without any augmentation methods (baseline) are the worst, with accuracy rates of only 84.24% and 50.00% on the two datasets. The implementation of RA or AA augmentation methods improves the results. Among them, AA achieves the best performance, with accuracy rates of 87.33% and 67.50% on the two datasets, while AA performs the poorest, with accuracy rates of 85.27% and 52.50%. Regarding AA augmentation, this is a resource-intensive method with a large search space of up to 1032. When applied to the two acne grading datasets with limited data, it often fails to realize its full potential. In contrast, AA is inspired by the effective search space of RA, offering a search space of only 102. Compared to the original RA, it excludes color-related augmentation methods (e.g., color) and methods that cause overlapping or confusion among lesion features (e.g., sample pairing), resulting in good performance in the experiments.

#### 4.5.4. Comparison Between Different Learning Structures

To validate the effectiveness of the AFF module proposed in this study, we conducted a comparison between AFF and other semi-supervised methods on the two acne grading datasets, ACNE04 and ACNE-ECKH. The commonality among the methods used in this work is that they all use EfficientNet-L2 as the backbone and do not employ any augmentation methods or modules. The difference lies in the training architecture, where the baseline represents the supervised method, and the remaining four are variations of semi-supervised methods.

In Table 12, the results for both acne grading datasets reveal that AFF achieves the best or second-best results in almost all metrics. This indicates that this method can train good models and improve the feature diversity. Compared to the second-best performer, MPL, it improves the loss function regarding the inter-class similarity and intra-class variance, causing the lesion features to cluster more effectively, resulting in the best performance in terms of precision, sensitivity, and accuracy. Interestingly, from the results of ACNE04, it is evident that the baseline achieves particularly high specificity. This is due to the supervised method converging more quickly to the lesion features in the labeled dataset, making its ability to diagnose negative cases stand out, which also elevates the YI. However, supervised learning is limited by the diversity of the lesion features in the dataset, making its ability to diagnose positive cases relatively poor. In contrast, semi-supervised methods can learn more lesion features from the unlabeled dataset, allowing the model to cope better with diverse acne lesions. Therefore, from the sensitivity or specificity results, it is evident that the model possesses a more robust ability to correctly diagnose positive and negative cases.

In summary, semi-supervised methods exhibit robust diagnostic capabilities compared to supervised methods. Among the four semi-supervised methods, AFF excels in its sensitivity results, demonstrating its effectiveness in handling inter-class similarity and intra-class variance, and it has the most comprehensive diagnostic capabilities.

## 5. Conclusions

Clinical image analysis faces persistent challenges in acne grading, including limited annotated data, the morphological ambiguity of lesions, complex pattern variations, and inconsistent diagnostic standards. To overcome these limitations, we propose the FF-PLL framework, integrating three core innovations: (1) the AFF structure, with self-correcting feedback loops for iterative pseudo-label refinement; (2) AFSS to eliminate non-lesion interference through multi-scale feature fusion; and (3) the AcneAugment (AA) strategy, simulating spectral, topological, and occlusion-based perturbations. This novel approach minimizes the dependence on labeled data, enhances the feature diversity, and improves the model’s generalization. Additionally, compared to state-of-the-art methods, the proposed FF-PLL framework achieves 6.87% higher accuracy on ACNE04 and a 30% improvement on ACNE-ECKH. Specifically, these results demonstrate the effectiveness of the proposed framework in handling multi-standard acne grading with limited labeled data.

Future work can focus on several key areas to further enhance the FF-PLL framework and its application in acne grading. First, integrating multimodal data, such as medical history and genomic information, alongside image data could improve the diagnostic accuracy. Second, expanding the data augmentation strategies to simulate varying lighting conditions, contrast enhancement, and additional occlusion types could increase the model’s robustness. Additionally, the integration of self-supervised and semi-supervised learning techniques could minimize the dependency on labeled data, enabling the model to learn effectively from limited annotations.

## Figures and Tables

**Table 1:** LinkNet encoder–decoder components.

Block (*i*)	Encoder (*m*, *n*)	Decoder (*m*, *n*)
1	(64, 64)	(64, 64)
2	(64, 128)	(128, 64)
3	(128, 256)	(256, 128)
4	(256, 512)	(512, 256)

**Table 2:** Comparison of the proposed schemes for the augmentation strategies.

Method	RA in CIFAR-10/ImageNet	RA in SVHN	AA in ACNE04/ACNE-EKCH
AutoContrast	0.1	0.1	0.1
Brightness	0.0	0.0	0.1
Color	0.0	0.0	0.0
Contrast	0.0	0.0	0.0
Equalize	0.1	0.1	0.3
Invert	0.1	0.1	0.0
Sharpness	0.1	0.1	0.0
Posterize	−0.3	−0.3	0.0
Solarize	−0.1	−0.1	0.0
Rotate	1.3	0.0	1.3
Shear-x	0.9	0.9	0.9
Shear-y	0.9	0.9	0.9
Shear-y	0.9	0.9	0.9
Translate-x	0.4	0.0	0.9
Translate-y	0.4	0.4	0.9
Sample pairing	0.1	0.0	0.0

**Table 3:** Number of samples for each grade in the two datasets: ACNE04 based on Hayashi and ACNE-ECKH based on Bernardis.

Dataset (Criterion)	ACNE04 (Hayashi)		ACNE-ECKH (Bernardis)	
Severity Level	Sample Quantity		Sample Quantity	
	Training	Test	Training	Test
0	410	103	34	10
1	506	127	216	10
2	146	36	194	10
3	103	26	16	10
Total	1165	292	460	40

**Table 4:** Comparison of two acne grading datasets.

Item		ACNE-ECKH	ACNE04
Data	Data Source (Institution)	Hospital (En Chu Kong Hospital, Taiwan)	Hospital (N/A), internet, others
	Digital Device	CANFIELD 6th VISIA; NIKON COOLPIX A1000; NIKON COOLPIX A900	N/A
	Shooting Method	Right/left/front face in each person	Confusion
	Images	8242	1457
	Objects	N/A	18,983
Right to Privacy	IRB (Institution)	Pass (En Chu Kong Hospital, Taiwan)	N/A
	De-Identification	Yes	No
	Association	MISA-TW; HIPAA; CoE 108	No
Patient	Age Range	18~52	N/A
	Skin Type	Fitzpatrick scale, II~IV	N/A
	People	Asian	Asian
Grading	Acne Grading (Reference)	0, 1, 2, 3 (Bernardis scale)	Mild, moderate, severe, very severe (Hayashi scale)
	Primary Lesions	Yes	Yes
	Secondary Changes	Yes	No
Label	Expert Labeled	Yes	Yes
	Expert Number	5	N/A

N/A: Not applicable.

**Table 5:** Hyperparameters in supervised learning experiments.

Hyperparameter	ACNE-ECKH	ACNE04
Weight decay	0.0005	0.0005
Batch normalization decay	0.99	0.99
Initial learning rate	0.01	0.01
Number of training steps	50,000	50,000
Number of warm-up steps	2500	2500
Batch size	8	16
Dropout rate	0.2	0.2
Pseudo-label threshold	0.95	0.95

**Table 6:** Hyperparameters in semi-supervised learning experiments.

Item	Hyperparameter	ACNE-ECKH	ACNE04
Common	Weight decay	0.0005	0.0005
	Label smoothing	0.1	0.1
	Batch normalization decay	0.99	0.99
	Number of training steps	3,000,000	1,000,000
	Number of warm-up steps	2000	2000
Student	Learning rate	0.3	0.3
	Batch size	8	8
	Dropout rate	0.35	0.35
Teacher	Learning rate	0.125	0.125
	Batch size	8	8
	Dropout rate	0.5	0.5
	UDA factor	1.0	1.0
	UDA temperature	0.8	0.8

**Table 7:** Comparison between different methods on ACNE04 dataset.

Type of Method	Method	Pre. ↑	Sp. ↑	Se. ↑	YI. ↑	Acc. ↑
Label distribution learning methods	PT-Bayes	45.31	79.39	45.06	24.44	45.38
	PT-SVM	44.60	83.04	46.05	29.10	48.15
	AA-KNN	67.61	87.73	67.33	55.05	68.15
	AA-BP	65.36	87.37	58.65	46.02	66.44
	SA-IIS	60.45	85.93	60.17	46.10	63.22
	SA-BFGS	73.85	91.01	72.03	63.03	76.16
	SA-CPNN	47.60	80.40	47.15	27.55	46.92
	DLDL [46]	78.51	92.24	78.57	68.81	79.31
Handcrafted feature methods	HOG [47]	39.10	77.91	38.10	16.01	41.30
	SIFT [48]	42.59	78.44	39.09	17.53	45.89
	GABOR [49]	45.35	79.89	41.78	21.67	48.22
	CH [50]	43.40	48.70	41.27	19.97	47.47
DL methods	SE-Res18	65.10	86.89	62.75	49.63	66.30
	SE-Res50	68.32	89.10	68.16	57.26	71.30
	EfficientNet-B0	72.16	90.46	69.00	59.45	75.07
	EfficientNet-B4	70.34	90.38	69.32	59.70	74.52
	EfficientNet-L2	78.42	91.56	77.43	69.71	80.46
	ResNet-50	72.25	90.82	71.13	61.95	75.89
	ResNet-152	70.34	89.10	70.11	61.95	74.42
	VGG-16	77.51	92.26	75.36	67.63	79.79
	LDL [18]	84.37	93.80	81.52	75.32	84.11
	KIEGLFN [51]	83.58	81.95	**94.11**	76.06	84.52
	EGPK [52]	84.01	**94.40**	84.62	**79.01**	85.27
	This work	**88.25**	90.14	87.31	77.45	**87.33**

**Table 8:** Comparison between different DL methods on ACNE04 and ACNE-ECKH datasets.

Dataset	ACNE04					ACNE-ECKH				
Method	Pre. ↑	Sp. ↑	Se. ↑	YI. ↑	Acc. ↑	Pre. ↑	Sp. ↑	Se. ↑	YI. ↑	Acc. ↑
SE-Res18	65.10	86.89	62.75	49.63	66.30	45.00	15.38	33.33	−51.29	27.50
SE-Res50	68.32	89.10	68.16	57.26	71.30	47.37	16.67	32.14	−51.19	27.50
EfficientNet-B0	72.16	90.46	69.00	59.45	75.07	50.00	16.67	35.71	−47.62	30.00
EfficientNet-B4	70.34	90.38	69.32	59.70	74.52	45.00	21.43	34.61	−43.96	30.00
EfficientNet-L2	78.42	91.56	77.43	69.71	80.46	65.00	22.22	41.93	−35.84	37.50
ResNet-50	72.25	90.82	71.13	61.95	75.89	44.44	16.67	44.44	−38.89	25.00
ResNet-152	70.34	89.10	70.11	61.95	74.42	47.36	9.09	47.36	−43.55	25.00
VGG-16	77.51	**92.26**	75.36	67.63	79.79	45.00	8.33	32.14	−59.52	25.00
This work	**88.25**	90.14	**87.31**	**77.45**	**87.33**	**70.00**	**68.42**	**66.66**	**35.08**	**67.50**

**Table 9:** Comparison between different modules on ACNE-ECKH dataset.

AFF	AFSS	AA	Acc. (%)	∆ (%)
✗	✗	✗	37.50	0
✓	✗	✗	42.50	+5.00
✓	✓	✗	50.00	+12.50
✓	✗	✓	47.50	+10.00
✓	✓	✓	67.50	+30.00

“✗” means the mechanism is not disable. “✓” means the mechanism is enable.

**Table 10:** Comparison between different modules on ACNE04 dataset.

AFF	AFSS	AA	Acc. (%)	∆
✗	✗	✗	80.13	0
✓	✗	✗	83.21	+3.08
✓	✓	✗	84.24	+4.11
✓	✗	✓	85.95	+5.82
✓	✓	✓	87.33	+7.20

“✗” means the mechanism is not disable. “✓” means the mechanism is enable.

**Table 11:** Comparison with some AA methods on different datasets.

Dataset	Search Space	ACNE04					ACNE-ECKH				
Method		Pre. ↑	Sp. ↑	Se. ↑	YI. ↑	Acc. ↑	Pre. ↑	Sp. ↑	Se. ↑	YI. ↑	Acc. ↑
Baseline	N/A	75.34	75.83	76.92	52.76	84.24	35.00	65.00	35.00	0.00	50.00
AA [53]	10^32^	75.34	79.42	**94.01**	73.44	85.27	50.00	52.38	52.63	5.01	52.50
RA^a^ [54]	10^2^	**89.04**	88.48	84.96	73.45	86.64	65.00	61.11	59.09	20.20	60.00
RA^b^ [54]	10^2^	88.35	87.76	84.31	72.08	85.95	60.00	57.89	57.14	15.03	57.50
AA (This work)	10^2^	88.25	**90.14**	87.31	**77.45**	**87.33**	**70.00**	**68.42**	**66.66**	**35.08**	**67.50**

RA^a^: RandAugment in CIFAR/ImageNet dataset. RA^b^: RandAugment in SVHN dataset. N/A: Not applicable.

**Table 12:** Comparison with some training methods on different datasets.

Dataset	ACNE04					ACNE-ECKH				
Method	Pre. ↑	Sp. ↑	Se. ↑	YI. ↑	Acc. ↑	Pre. ↑	Sp. ↑	Se. ↑	YI. ↑	Acc. ↑
Baseline	78.42	91.56	77.43	**69.71**	80.46	65.00	22.22	41.93	−35.84	37.50
Noisy Student [38]	82.19	82.06	81.63	63.70	81.84	70.00	14.28	**42.42**	−43.29	37.50
UDA	79.45	80.00	81.69	61.69	80.82	55.00	30.76	40.74	−28.49	37.50
MPL [42]	82.87	**86.82**	79.14	65.96	82.53	55.00	**35.71**	42.30	−21.97	40.00
AFF (This work)	**88.35**	82.99	**83.44**	66.44	**83.21**	**70.00**	33.33	45.16	**−21.50**	**42.50**

## Data Availability

As ethical, legal, or privacy issues are present, the data cannot be shared. It is necessary to ensure that the publication of such data does not compromise the anonymity of the participants or breach local data protection laws. Those who plan to engage in related research in the future can contact the corresponding author, Dr. Chii-Shyan Wang, at En Chu Kong Hospital.
